# Development of Single-Channel Hybrid BCI System Using Motor Imagery and SSVEP

**DOI:** 10.1155/2017/3789386

**Published:** 2017-08-07

**Authors:** Li-Wei Ko, S. S. K. Ranga, Oleksii Komarov, Chung-Chiang Chen

**Affiliations:** ^1^Brain Research Center, National Chiao Tung University, Hsinchu City, Taiwan; ^2^Institute of Bioinformatics and System Biology, National Chiao Tung University, Hsinchu City, Taiwan; ^3^Department of Biological Science and Technology, National Chiao Tung University, Hsinchu City, Taiwan; ^4^Institute of Molecular Medicine and Bioengineering, National Chiao Tung University, Hsinchu City, Taiwan; ^5^Office of Physical Education, National Chiao Tung University, Hsinchu City, Taiwan

## Abstract

Numerous EEG-based brain-computer interface (BCI) systems that are being developed focus on novel feature extraction algorithms, classification methods and combining existing approaches to create hybrid BCIs. Several recent studies demonstrated various advantages of hybrid BCI systems in terms of an improved accuracy or number of commands available for the user. But still, BCI systems are far from realization for daily use. Having high performance with less number of channels is one of the challenging issues that persists, especially with hybrid BCI systems, where multiple channels are necessary to record information from two or more EEG signal components. Therefore, this work proposes a single-channel (C3 or C4) hybrid BCI system that combines motor imagery (MI) and steady-state visually evoked potential (SSVEP) approaches. This study demonstrates that besides MI features, SSVEP features can also be captured from C3 or C4 channel. The results show that due to rich feature information (MI and SSVEP) at these channels, the proposed hybrid BCI system outperforms both MI- and SSVEP-based systems having an average classification accuracy of 85.6 ± 7.7% in a two-class task.

## 1. Introduction

A brain-computer interface (BCI) establishes a human-to-device communication channel by translating the brain signals into machine codes to control external devices or applications [1, 2]. Over the decade, unprecedented advances were made in the field of BCI trying to bring laboratory studies to real-world applications [3]. Numerous techniques for feature extraction methods [4–6], classification algorithms [7, 8], and experimental paradigms [9, 10] have been developed. The majority of these systems were based on a single modality of EEG, that is, they either use motor imagery (MI) [9], P300 [10], or steady-state visually evoked potential (SSVEP) [11]. Recently, hybrid BCI systems started gaining importance due to their promising benefits in terms of classification accuracy or the number of user commands available for the user [12, 13]. A hybrid BCI system is a combination of a primary BCI system with another communication channel, which can be a BCI or another system based on a physiological signal recognition like electromyography (EMG) and electrooculography (EOG). Examples of EEG-based hybrid BCI systems include MI + SSVEP [14–17], SSVEP + P300 [18, 19], and SSVEP + EMG [20]. It has been shown that it is even possible to create a vision-independent hybrid BCI system combining an auditory and tactile P300 recognition [21]. In this study, we focus on technological progression of a hybrid BCI system using MI and SSVEP.

Although SSVEP is an efficient BCI method with a variety of applications, adding MI features is a convenient way to improve it, since an implementation of MI does not require providing of any additional visual stimulus to the operator, just a cognitive task. In recent years, a few studies made a significant contribution towards the hybrid BCI developments combining MI and SSVEP: a five-channel hybrid system utilized C3, Cz, C4, O1, and O2 channels [22], and a four-channel hybrid system utilized C3, C4, O1, and O2 [23]. The choice of both central and occipital regions is based on the fact that EEG signals from the motor and visual cortices have to be processed to extract MI and SSVEP features, respectively. Despite their high accuracy and no need for special training, SSVEP-based BCI systems are strongly limited to the need for the placement of EEG electrodes in the occipital area, which can be problematic in some real cases. For example, it may happen if a BCI operator is lying face up, sits in a seat with a headrest, or the used EEG system simply does not have electrodes in the occipital area. It has been shown that it is possible to measure SSVEP signal even from non-hair-bearing areas [24], but the degraded signal-to-noise ratio can be an obstacle for the development of an accurate BCI. In our previous work, we demonstrated that SSVEP information could also be extracted from central EEG channels [25, 26] and addition of MI features was an effective way to compensate the declined SSVEP performance [27]. As it is important to obtain good BCI accuracy, it is also necessary to have a few channel systems for practical use depending on the application. It has been demonstrated that MI-related features can be extracted from a single EEG channel using short-time Fourier transform (STFT) and common spatial pattern (CSP) [28].

With these developments, this work aims to develop a hybrid BCI system by adopting a combination of MI and SSVEP using a single channel from the central region (either C3 or C4). For EEG recording, subjects were asked to perform three different tasks, namely, (1) right hand (RH) and left hand (LH) MI, (2) visually focus on 15 Hz or 20 Hz SSVEP flicker, and (3) perform MI and SSVEP simultaneously. Due to the presence of both MI's event-related desynchronisation (ERD) feature and SSVEP's prominent peak at the flicker frequency, the hybrid BCI is expected to show better performance in comparison with a single mode BCI. Details of the experimental design are further discussed in Section 2. In the proposed single-channel system, we applied the short-time Fourier transform (STFT) and common frequency pattern (CFP) method for feature extraction. Linear discriminant classifier (LDC) was used to estimate the classification accuracy. Results show that due to the rich of feature information from central channels in the hybrid condition, the proposed hybrid BCI system can achieve high classification accuracy of 85% utilizing a single EEG channel. See Section 3 for more details on the results.

## 2. Materials and Methods

### 2.1. Participants

Seventeen subjects (12 male, age: 23.1 ± 2.6 years) with no history of any health ailments were recruited for this study. All the subjects have normal or corrected to normal vision. Each participant was informed of the experimental procedure, and a written consent form was taken. All the participants had no prior experience with BCI. The experiment was performed in accordance with the country's laws and approved by the institutional review board (IRB) of the National Chiao Tung University (NCTU), Hsinchu, Taiwan.

### 2.2. Experiment Paradigm

**Figure 1:**
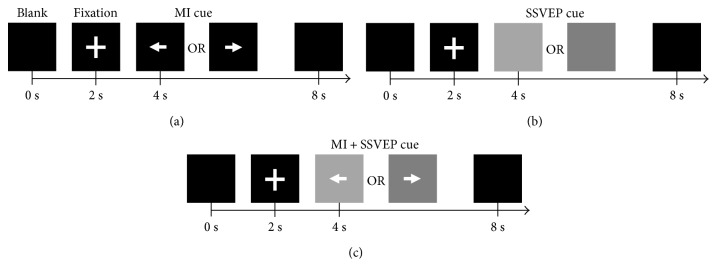
The experimental paradigm for (a) MI task, (b) SSVEP task, and (c) hybrid (MI + SSVEP) task. Only one visual target appeared on the screen in a single trial. The gray boxes represent 20 Hz (light gray, left) and 15 Hz (dark gray, right) flickering black/white stimuli for SSVEP induction. The subjects were instructed to perform the tasks constantly while the cues were displayed.

During the experiment, each of the participants of this study was seated in a comfortable position and performed three different tasks as follows:

1. MI task: This task consists of two classes, left hand- and right hand-imagined movements. After initial training, three sessions of MI data were recorded. In each session, 15 trials per class were recorded. Combining three sessions, this task comprises of 45 trials per class. See Figure 1(a) for the paradigm overview of this task. For MI stimulus, a left cue was presented as an indication for the subject to perform the left hand MI and a right cue for the right hand MI.
2. SSVEP task: In this task, the subjects were asked to focus visually on a flickering black/white stimulus presented on the screen (21″ LCD, 60 Hz refresh rate, 1920 × 1080 screen resolution). The frequencies used for the stimulus presentation were 15 Hz and 20 Hz (see Figure 1(b)). Three sessions of SSVEP data were recorded, with each session containing 10 trials per class.
3. Hybrid task: In this task, the subjects were instructed to focus visually on the flickering SSVEP stimulus and simultaneously perform the right hand (RH) MI or the left hand (LH) MI. The two classes include the following: (1) RH-MI + 15 Hz-SSVEP and (2) LH-MI+ 20 Hz-SSVEP. See Figure 1(c) for the overview of this task. A left cue was presented (which was programmed to flicker at 20 Hz) to indicate to the user to perform LH MI and fixate on the flicker. Similarly, a right cue (flickering at 15 Hz) indicated to the users to perform RH MI and fixate on the stimulus. Three sessions of hybrid task were recoded for each subject, with each session comprising 15 trials per class.

All the above tasks followed the similar procedure with a blank for 2 seconds, then a fixation (indicated by plus sign) for 2 seconds, and a stimulus cue for 4 seconds. The subjects were asked to perform a task during the entire duration of the cue display. No feedback was provided to the subjects during the experiment, and all analyses were done offline. Each subject performed the three above-mentioned tasks in a random order.

### 2.3. Data Acquisition and Preprocessing

**Figure 2:**
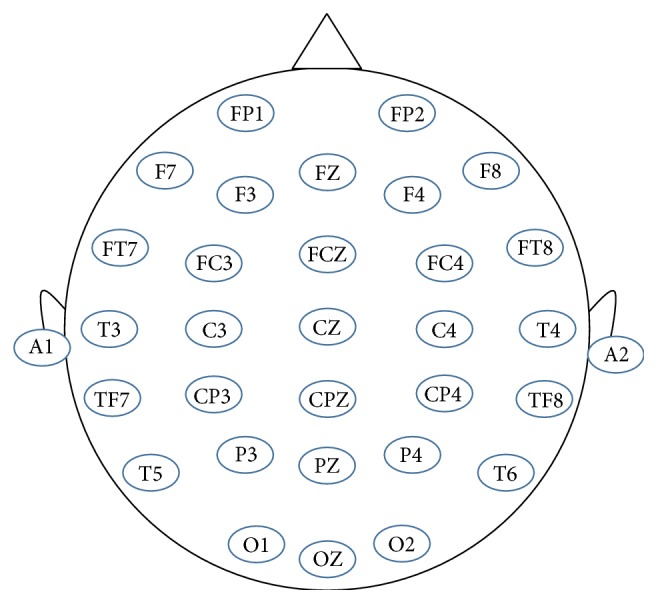
32 EEG electrodes were placed according to the International 10–20 system. A1 and A2 reference electrodes were located on the earlobes.

A 32-channel (with EEG electrodes placed according to the 10–20 international system, see Figure 2) system from Neuroscan was used in this study for EEG recording. The data were acquired at a sampling rate of 500 Hz, and the impedances of all the channels were kept below 5 kΩ. All the preprocessing and analysis steps were done offline in MATLAB 2014b and using EEGLAB [29], a MATLAB toolbox for EEG data processing. The recorded EEG data were filtered using a 1~50 Hz bandpass filter to remove power line noise (60 Hz) and other high-frequency noises. And then, epochs were extracted for further analysis.

### 2.4. Short-Time Fourier Transform (STFT)

STFT divides a signal (channel data) into many segments and then computes the Fourier transform for each segment individually. With STFT, the time series signal from an EEG channel was transformed into time-frequency domain with a window segment size of 500 ms and an overlap of 250 ms. MATLAB function spectrogram from signal processing toolbox was used for implementing the STFT.

### 2.5. Common Frequency Pattern (CFP)

Common spatial pattern or CSP is a well-known algorithm for MI feature extraction [30]. CSP finds an optimal spatial pattern from the time series EEG signal, and these spatial patterns are responsible for distinguishing two classes. Similar to CSP, CFP focuses on optimal frequency bands for distinguishing the two classes based on the frequency domain data [26, 31]. In our previous work, we have implemented CFP taking power spectrum (PSD) as the input [25, 26]. Normally, the PSD obtained from multiple channels (forming a 2D matrix) is taken as an input for CFP. However, a single-channel system lacks the ability to form a 2D matrix by adopting PSD. Implementing STFT on single-channel time series data can output a 2D matrix containing time-frequency information, which is a plausible input for CFP. The features obtained by CFP are then used for classification purpose to estimate the system's performance. The algorithm for CFP-based feature extraction is as follows.

Covariance of the time-frequency data (E) is calculated as follows for each trial:

(1) $$\begin{array}{c} C=\frac{E^{'}E}{N}. \end{array}$$

A composite covariance is estimated by summing the covariance of each group (averaged across all the trials in a group):

(2) $$\begin{array}{c} C_{c}={\overset{-}{C}}_{l}+{\overset{-}{C}}_{r}. \end{array}$$

A whitening transformation is applied such that all the eigenvalues of *PC*_*c*_*P*′ are equal to one, where *U*_*c*_ and *λ*_*c*_ are the eigenvector and eigenvalues of *C*_*c*_, respectively, and $P=\sqrt{\lambda_{c}^{-1}}{U^{\prime }}_{c}$.

$\overset{-}{C_{l}}$ and *C*_*r*_ are transformed to *S*_*l*_ and *S*_*r*_, and they share common eigenvectors as follows:

(3) $$\begin{array}{c} S_{l}=P{\overset{-}{C}}_{l}P^{\prime },S_{r}=P{\overset{-}{C}}_{r}P^{\prime } \end{array}$$

(4) $$\begin{array}{c} S_{l}=B\lambda_{l}B^{\prime },S_{r}=B\lambda_{r}B^{\prime },\lambda_{l}+\lambda_{r}=I. \end{array}$$

The sum of *λ*_*l*_ and *λ*_*r*_ is equal to one. The eigenvector with the largest eigenvalue for *S*_*l*_ will have the smallest eigenvalue for *S*_*r*_ and vice versa. A new trial's time-frequency data (E) is mapped with the projection matrix as follows:

(5) $$\begin{array}{c} Z=WE,where\;W=P^{'}B. \end{array}$$

The first and last filters from *W* provide maximum variance for one class and the lowest variance for the other class. The feature vector *f*_*cfp*_ is calculated as follows:

(6) $$\begin{array}{c} f_{cfp}=\frac{\text{diag}\left(\text{cov}\left(Z{'}_{p}\right)\right)}{N}. \end{array}$$

The signals *Z*′*p* (*p* = 1, 2,…, 2*m*) is formulated by selecting the first *m* and last *m* filters that maximize covariance's difference between the two classes. The feature vectors obtained with CFP will be passed into the classification stage for estimating the BCI system performance.

**Figure 3:**
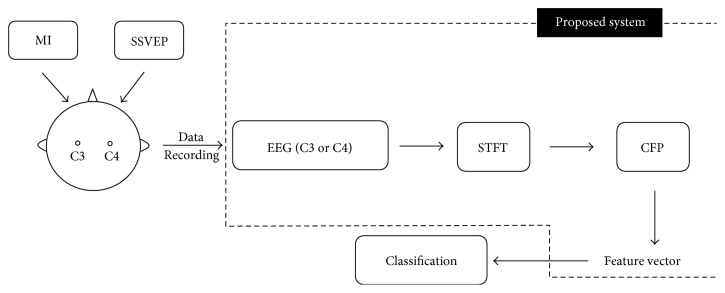
Flowchart of EEG data acquisition and processing in the proposed single-channel (C3 or C4) hybrid BCI system.

The framework of the proposed hybrid BCI system is shown in Figure 3. In this work, all the analyses and results are based on a single channel, using either C3 or C4 channel.

### 2.6. Classification

LDC classifier was applied to estimate classification accuracies in the implemented tasks using 5-fold cross validation. In each fold, the training data were used for generating a weight matrix by CFP, and then it was applied on the testing data for generating the test features. PRtool 5, a MATLAB toolbox for pattern recognition, was used for the classification purpose. A paired *t*-test was applied to validate significant differences in the performances among the proposed hybrid BCI system with different parameters, MI-BCI, and SSVEP-BCI. The classification accuracies of different BCI modalities were estimated using EEG data recorded from their respective tasks. For example, the MI-BCI accuracy was estimated using the data recorded from the MI task.

## 3. Results

### 3.1. Power Spectrum

**Figure 4:**
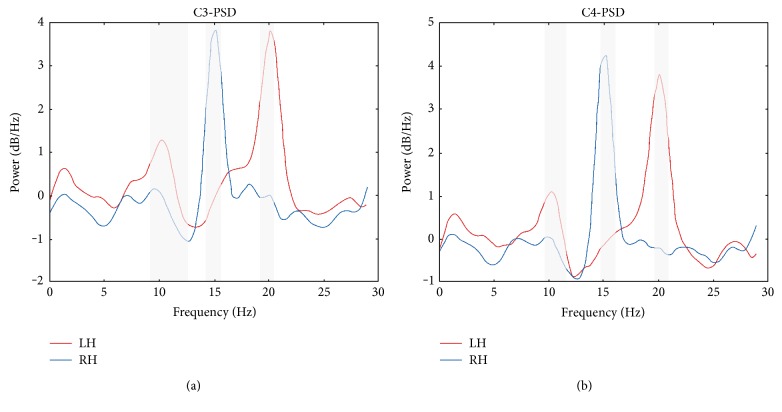
Averaged power spectrum of the hybrid EEG signal at (a) C3 channel and (b) C4 channel. The gray regions represent significant differences in the PSD (*p* < 0.05).

Figure 4 shows the averaged (of all subjects) power spectral density of the hybrid task's data. EEGLAB's function spectopo was used for generating the PSDs. The legend LH and RH indicates the left hand MI + 20 Hz SSVEP and the right hand MI+ 15 Hz SSVEP cued trials of the hybrid task. Due to the simultaneous performance of both MI and SSVEP during this task, MI- (ERD feature) and SSVEP- (dominant peak at stimulus frequency) related spectral patterns can be explicitly observed from the data recorded over central channels C3 and C4.

### 3.2. Classification Accuracy in Different Tasks

**Figure 5:**
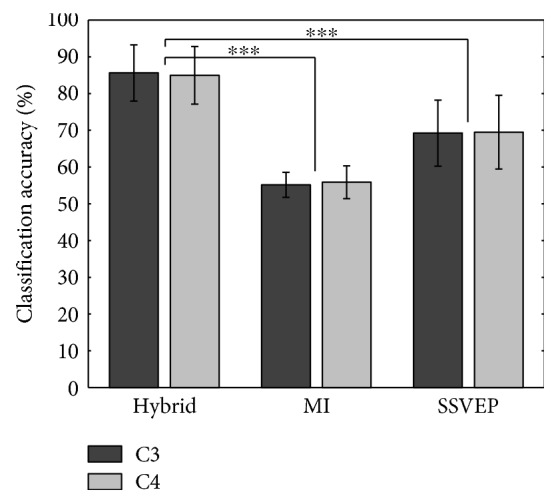
Mean ± std classification accuracy in the proposed hybrid, MI, and SSVEP BCIs using LDC for C3 and C4 channels (^∗∗∗^*p* < 0.001).

The classification accuracy was estimated for C3 and C4 channels separately and independently from each other. For C3 channel, the highest average accuracy of 85.62 ± 7.67% was obtained in the hybrid task, whereas, the accuracy was 55.15 ± 3.38% in the MI task and 69.22 ± 8.98% in the SSVEP task. Similarly, for C4 channel, the hybrid task resulted in 84.98 ± 7.85%, whereas, an accuracy of 55.87 ± 4.48% was reached in the MI task and 69.47 ± 10.04% in the SSVEP task. The performance of the hybrid BCI system is significantly higher (see Figure 5) in comparison to MI-BCI (*p* < 0.001, paired *t*-test) and SSVEP-BCI (*p* < 0.001, paired *t*-test). Also, no significant difference has been observed between the performance of C3 channel- and C4 channel-based hybrid BCI systems, leading to a logical conclusion that the proposed single channel hybrid BCI system can work with either C3 or C4 channel.

### 3.3. Classification Accuracy and Training Dataset Size

**Figure 6:**
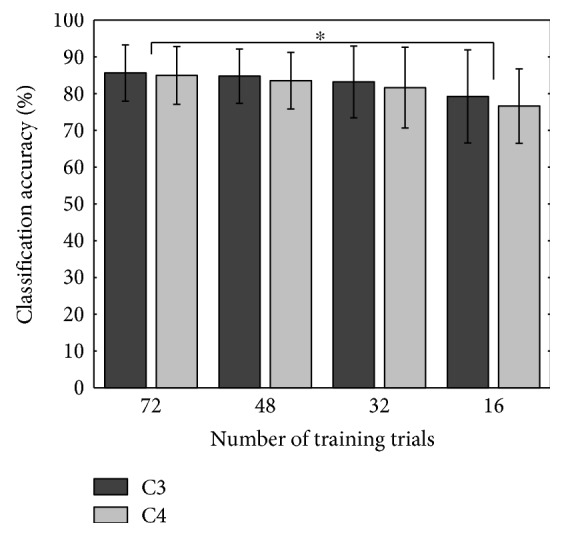
Mean ± std classification accuracy of the hybrid BCI system using different numbers of trials for the classifier training (^∗^*p* < 0.05).

For any BCI system, it is usual for the performance to decrease when fewer trials are available for the classifier training. To test the stability of the proposed hybrid BCI approach, the number of trials in training datasets for the cross-validation was reduced to 48, 32, and 16 trials, which simulates 5-fold classifications using 30, 20, and 10 trials per class, respectively. The average classification accuracy significantly decreases (*p* < 0.05, paired *t*-test) from 85.62 ± 7.67% and 84.98 ± 7.58% to 79.25 ± 12.67% and 76.63 ± 10.12% for C3 and C4 channels, respectively, when 16 trials are used for the classifier training instead of 72 (see Figure 6()). No significant differences in the classification accuracy have been observed using 48 or 32 trials for the classifier training as compared with 72 trials.

### 3.4. Classification Accuracy and Trial Duration

**Figure 7:**
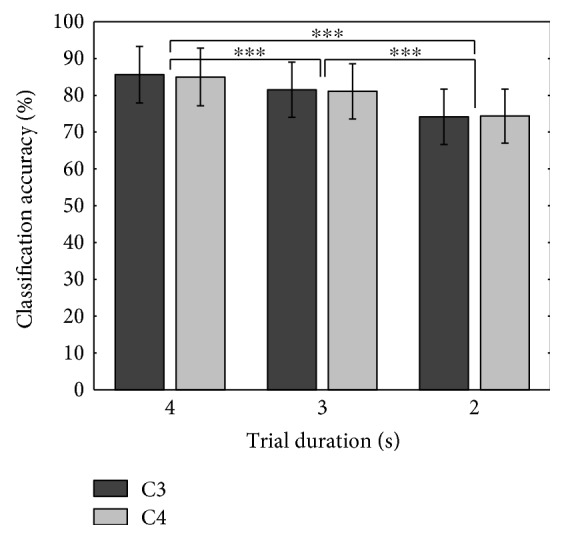
Mean ± std classification accuracy of the hybrid BCI system for different trial durations using LDC for C3 and C4 channels (^∗∗∗^*p* < 0.001).

Longer EEG samples allow a BCI system to achieve higher performance, extracting more information about the brain activities, but make it difficult to operate the system in real time. To evaluate the system's accuracy with different trial durations, different time window segments were extracted from the recorded original trials with the initial point set to the beginning of a stimulus onset. For example, 2 s trial duration means that a two-second wide time window spans across the initial two seconds (starting from the stimulus onset).  Figure 7 shows variations in the proposed system's accuracy depending on different trial durations. As the trial duration decreases from 4 s to 2 s, the classification accuracy also decreases significantly from 85.62 ± 7.67% to 74.14 ± 7.53% at C3 channel and 84.98 ± 7.58% to 74.36 ± 7.34% at C4 channel.

## 4. Discussion and Conclusion

**Figure 8:**
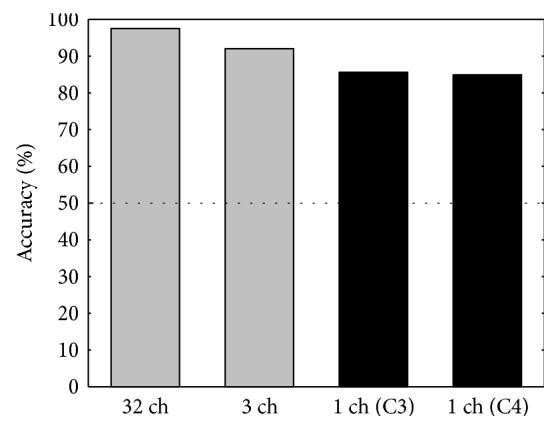
Classification accuracies of 32-, 3- (from [26]), and single-channel MI + SSVEP hybrid systems in a two-class task. The black bars represent the results of this study. The three- and single-channel systems utilize EEG electrodes located in the central area (C3-Cz-C4, C3, and C4, resp.).

This study demonstrates a transition of hybrid BCI systems towards a reduction of the number of EEG channels. In our past work [26], two different multichannel systems with 3 and 32 EEG channels were developed; both of them can outperform the proposed system in terms of accuracy (Figure 8). However, there is always a trade-off between the classification accuracy and the number of electrodes used in a system. More EEG channels allow us to perform better feature extraction and consequently reach higher accuracy. However, single-channel systems are convenient and suit the purpose of practical daily life applications being an important topic for further research. Combining SSVEP and MI modalities to a hybrid BCI, it becomes possible to compensate the declined performance. In addition to that, hybrid systems can compensate the BCI illiteracy issue, when some people cannot effectively use a particular modality of BCI. With a hybrid system (MI + SSVEP), a user with MI-BCI illiteracy could still use the system just by using SSVEP alone, which increases its universality. In our previous study [27], we compared the performance of an SSVEP system utilizing two pairs of EEG channels from the central and occipital areas. The classification accuracy of the system in a two-class task was 89.94 ± 3.94% with O1-O2 channels and 85.53 ± 2.69% with C3-C4 channels. The proposed, in this study, hybrid system demonstrates a comparable level of classification accuracy combining important advantages of utilizing just a single EEG channel and providing more freedom in the channel placement as compared with a single-mode SSVEP-based BCIs.

It has been shown that it is possible to induce an SSVEP response in a wide frequency range [32] starting from very low frequencies [33]. In this study, we demonstrate the presence of both MI- and SSVEP-related changes in the PSD during performing a combined MI + SSVEP cognitive task. Based on spectral characteristics of the MI and SSVEP responses, it is reasonable to use high frequencies for the SSVEP stimulus in MI + SSVEP hybrid systems to avoid their overlapping (Figure 4). The MI approach requires from the user to perform intentionally a cognitive task, which means that the MI response appearance is delayed regarding the MI cue onset depending on the reaction time [34]. As a result, reducing the duration of a trial beyond a certain limit may decrease the contribution of MI features to the hybrid BCI performance.

The major limitation of the proposed and tested system is a few number of classes, that is, only two output commands are available for a user's BCI application. Therefore, the potential future work has to be focused on expanding the number of user commands but retaining the priority of a single-channel system and optimal performance.

## Supplementary Material

Development of Single Channel Hybrid BCI System using Motor Imagery and SSVEP
