# Supplementary material for: Development of Single-Channel Hybrid BCI System Using Motor Imagery and SSVEP
Source: J Healthc Eng. 2017 Aug 7;2017:3789386. doi: 10.1155/2017/3789386 (PMC5564129; doi:10.1155/2017/3789386)
Supplement: Supplementary file 1 — Development of Single Channel Hybrid BCI System using Motor Imagery and SSVEP [file 3789386.f1.pdf]

Development of Single Channel Hybrid BCI System using Motor Imagery and SSVEP

Subject wise accuracy tables

1) Classification accuracy for three tasks (hybrid, MI and SSVEP) at C3 channel using LDC (for duration of 4sec).

subject	Hyb	MI	SSVEP
1	95.86	54.14	76.50
2	72.88	53.41	61.50
3	74.78	52.00	68.50
4	90.82	55.75	52.50
5	83.89	54.67	64.00
6	92.75	60.82	60.00
7	89.41	60.56	58.50
8	72.94	49.56	67.00
9	89.14	61.18	71.00
10	94.38	54.71	76.50
11	92.82	53.22	69.50
12	89.38	53.43	64.50
13	90.88	51.78	77.50
14	77.25	55.88	70.50
15	78.86	52.50	80.50
16	83.88	58.88	89.00
Avg	85.62	55.15	69.22

2) Classification accuracy for three tasks (hybrid, MI and SSVEP) at C4 channel using LDC (for duration of 4sec).

subject	Hyb	MI	SSVEP
1	96.00	57.57	87.00
2	75.25	59.88	59.50
3	78.33	57.22	84.50
4	77.65	52.63	57.50
5	92.67	49.33	68.50
6	88.25	53.29	66.50
7	83.18	59.78	54.50
8	76.12	50.00	82.00
9	90.29	59.06	66.50

10	93.63	60.86	71.00
11	97.65	53.67	65.50
12	84.50	65.43	67.00
13	93.25	48.78	80.50
14	73.50	57.75	59.50
15	79.00	53.75	61.00
16	80.38	54.88	80.50
Avg	84.98	55.87	69.47

3) Classification accuracy of Hybrid task at C3 channel using LDC (for duration of 4, 3 and 2 seconds).

subject	4s	3s	2s
1	95.86	90.14	87.57
2	72.88	72.25	64.25
3	74.78	66.67	65.67
4	90.82	80.35	69.18
5	83.89	84.56	66.44
6	92.75	91.00	81.13
7	89.41	86.12	72.71
8	72.94	67.76	63.76
9	89.14	84.14	78.43
10	94.38	90.50	80.00
11	92.82	90.82	83.18
12	89.38	81.50	77.00
13	90.88	86.13	78.63
14	77.25	72.88	66.75
15	78.86	73.86	68.86
16	83.88	85.63	82.63
Avg	85.62	81.52	74.14

7) Classification accuracy of Hybrid task at C4 channel using LDC (for duration of 4, 3 and 2 seconds).

subject	4s	3s	2s
1	96.00	95.29	89.14
2	75.25	74.38	68.50
3	78.33	73.56	68.56
4	77.65	77.06	68.24
5	92.67	90.11	83.67
6	88.25	81.00	79.00

7	83.18	80.82	64.71
8	76.12	72.24	67.41
9	90.29	86.57	72.43
10	93.63	85.75	78.88
11	97.65	95.53	83.06
12	84.50	74.63	70.88
13	93.25	84.50	81.88
14	73.50	68.25	63.88
15	79.00	75.71	72.57
16	80.38	82.00	77.00
Avg	84.98	81.09	74.36
